# Directional Deep Brain Stimulation Programming in Parkinson's Disease and Essential Tremor Patients: An Institution-Based Study

**DOI:** 10.7759/cureus.89346

**Published:** 2025-08-04

**Authors:** Maria C Moreno-Escobar, Robin Elkins, Ann Murray, Nicholas Brandmeir, Cheryl Brandmeir, Srivatsan Pallavaram, Richa Tripathi

**Affiliations:** 1 Neurology, West Virginia University, Morgantown, USA; 2 Rockefeller Neuroscience Institute, West Virginia University, Morgantown, USA; 3 General Internal Medicine, West Virginia University, Morgantown, USA; 4 Neurosurgery, West Virginia University, Morgantown, USA; 5 Human Performance - Physical Therapy, West Virginia University, Morgantown, USA; 6 Neuromodulation, Abbott Laboratories, Austin, USA; 7 Neurology, Emory University, Atlanta, USA

**Keywords:** deep brain stimulation, directional programming, essential tremor (et), neuromodulation therapy, parkinson's disease

## Abstract

Deep brain stimulation (DBS) and the use of directional subsegmental stimulation have significantly advanced symptom management in patients with Parkinson’s disease (PD) and essential tremor (ET). This study examines the use of directional programming in a tertiary care center. We retrospectively reviewed medical records of 12 PD patients (all with bilateral subthalamic nucleus (STN) implants) and 13 ET patients (12 with bilateral and 1 with unilateral ventral intermediate nucleus (VIM) implants) who received directional leads. Clinical parameters such as the Fahn-Tolosa-Marin (FTM) Tremor Rating Scale for ET and the Unified Parkinson's Disease Rating Scale (UPDRS) for PD were assessed before and after the procedure. Programming parameters and the reasons for switching to directional stimulation were also evaluated. Directional programming was used in 11 of 12 (92%) PD patients and 12 of 13 (92%) ET patients on at least one side. Among PD patients, 58% (7/12) used directional programming to avoid side effects, while 25% (3/12) used it to improve therapeutic benefit. Similarly, among ET patients, 64% (7/11) used directional programming to avoid side effects, and 33% (4/12) experienced improved tremor control with directional configurations. Our study demonstrates high utilization of directional programming. The findings suggest directional stimulation improves efficacy by reducing side effects and enhancing therapeutic outcomes. These results may support the early adoption of directional programming in managing PD and ET patients.

## Introduction

Deep brain stimulation (DBS), a form of neuromodulation, has revolutionized the treatment of Parkinson's disease (PD), dystonia, essential tremor (ET), and refractory Tourette syndrome over the past 30 years [1-5]. Over time, DBS therapy has advanced to incorporate adjustments in current, pulse width, and frequency across multiple lead contacts to optimize stimulation delivery. Lead designs have also evolved to include subsegments, enabling individualized current delivery and enhanced flexibility in electric field modulation [6]. Despite these advancements, the side effect profile of DBS can limit its clinical benefit, patient tolerability, and overall success [5,7,8]. The development of subsegmental (directional) stimulation aims to minimize stimulation-induced side effects [6,7,9]. This technique allows for higher stimulation thresholds, potentially expanding the therapeutic window, and offers more precise control over the targeted tissue, especially useful in compensating for anatomical variations or minor deviations from the planned lead trajectory [1,6,9].

In this study, we evaluated the institutional use of directional lead programming in ET patients (implanted in the ventral intermediate nucleus (VIM) of the thalamus) and PD patients (implanted in the subthalamic nucleus (STN)). We assessed its impact on outcomes and examined the clinical reasons prompting a switch from omnidirectional (full-ring/segment) to directional stimulation, with the goal of identifying common triggers for this programming choice.

## Materials and methods

Data collection

A retrospective chart review was conducted for all patients diagnosed with PD and ET who underwent DBS surgery. Institutional Review Board (IRB) approval was obtained prior to data collection. Patients included in the study had implantation of directional Abbott leads, either SJM 6173 (1.5 mm spacing) or SJM 6172 (0.5 mm spacing). Relevant clinical information was reviewed, including age, gender, pre- and post-surgical UPDRS Part III motor scores (assessed in the on-medication state pre-surgery and on-medication/on-stimulation state post-surgery), pre- and post-surgical levodopa equivalent dose (LED), and the Fahn-Tolosa-Marin (FTM) tremor rating scale. Inclusion criteria included (1) diagnosis of PD or ET confirmed by a movement disorder specialist and (2) completion of at least two months of DBS programming for optimal clinical management.

Initial programming

At our institute, the initial programming visit includes a detailed evaluation of each contact, with assessment of both symptom control and the occurrence of side effects. Programming typically begins with standardized parameters: a frequency of 130 Hz, a pulse width of 60 microseconds, and variable current adjusted based on clinical response. Both therapeutic benefit and the presence or absence of side effects are carefully monitored. The side effect threshold is defined as the stimulation level below which no persistent neurological side effects are observed. 

Surgical technique

**Figure 1:**
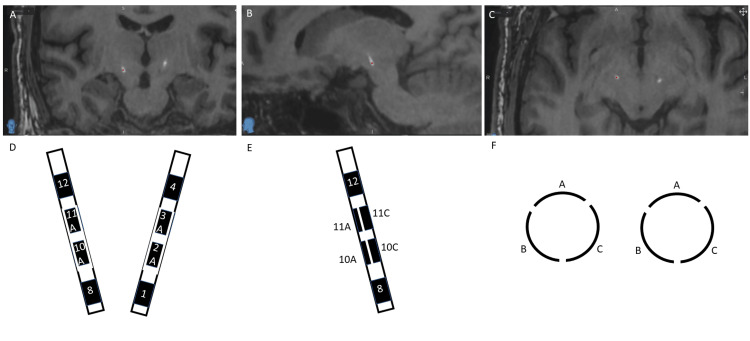
Schematic orientation of directional leads (A-C) Coronal, sagittal, and axial MRI-CT fused reconstructions showing subthalamic lead placement.  (D-F) Schematic representations of segmented lead orientation in the coronal, sagittal, and axial planes, respectively.  Schematics are not to scale.

All patients were evaluated by a multidisciplinary movement disorder clinic and deemed appropriate candidates for DBS surgery. Details of the surgical technique have been described previously [10]. With regard to the orientation of the directional leads, the “A” contact was consistently positioned anteriorly, regardless of implant laterality. The lead was measured so that the tip of the deep ring contact (contact “1”) would be located at the floor of the target nucleus, as determined by intraoperative microelectrode recordings. To minimize lead rotation, the lead was oriented such that the set screw of the lead carrier was placed to the right of the “A” contact. The distal portion of the lead was allowed to rotate freely within the cannula to achieve the planned orientation before insertion into the brain. The STN was targeted indirectly using coordinates relative to the mid-commissural point: 12 mm lateral, 3 mm posterior, and 6 mm inferior (Figure 1). Results of intraoperative testing and microelectrode recordings are summarized in Table 1.

**Table 1:** Intraoperative microelectrode recording and test stimulation data Tracks are defined relative to the standard Ben-Gun array. All measurements are in millimeters (mm), unless otherwise specified. Negative values indicate positions superficial to the planned target along the electrode's Z-axis. VIM: ventral intermediate nucleus of the thalamus, STN: subthalamic nucleus, MER: microelectrode recordings.

Patient	Target	Left			Right		
		MER results	Testing results	Final position	MER results	Testing results	Final position
1	STN	Center track (-)6-0	No side effects	Center track 1	Medial track (-)6-0	No side effects	Medial track 1.5
2	STN	Center track (-)3.5- 2	Transient paresthesias	Center track 2	Center track (-)5 – (-)2	Transient paresthesia	Center track 1
3	STN	Center track (-)6.5 – (-)1.5	No side effects	Center track (-)1.5	Center track (-)6.5 – (-)3	Speech slur 3mA	Center track (-)3
4	STN	Center track (-)3.36 - 0.36	Transient paresthesias	Center track 0.36	Center track (-)3 – 3	No side effects	Center track 3
5	STN	Center track (-)5 – 0	No side effects	Center track 0.5	Center track (-)5 – 0	No side effects	Center track 0
6	STN	Medial track (-)3 – 0	No side effects	Medial track 0	Center track (-)2 – 2	No side effects	Center track 2
7	STN	Center track (-)6 – 0	No side effects	Center track 0.5	Center track (-)1.5 – 1	Transient paresthesias	Center track 1
8	STN	Center track (-)5-0	No side effects	Center track 0	Posterior track (-)5.5- 0	No side effects	Posterior track 0
9	STN	Center track (-)4.5 – 0	No side effects	Center track 1	Center track (-)3 – 2	No side effects	Center track 3
10	NA	No data	No data	No data	No data	No data	No data
11	STN	Center track (-)5 – (-)1.5	Lip pulling @ 3mA	Center track (-)1	Posterior-Medial track (-)5.5) – (-)1.5	Lip pulling @ 3mA	Posterior-medial track (-1)
12	STN	Center track (-)5.5 – (-)1	No side effects	Center track (-)0.5	Center track (-)3.5-0	Transient paresthesias	Center track 2

Essential tremor							
Patient	Target	Left			Right		
		MER results	Testing results	Final Position	MER results	Testing Results	Final Position
1	VIM	Center track (-)5.5 – 0	Transient paresthesias	Center track 0	Center track (-)7.5-(-)3	Persistent paresthesias @ 3mA	Center track (-)3
2	VIM	Center track (-)8.5-0	No side effects	Center track 0	Center track (-)8 – 1	No side effects	Center track (-)0.5
3	VIM	Center track (-)7-0	No side effects	Center track 0	Center track (-)8-0.5	No side effects	Center track 0.5
4	VIM	Center track (-)6.5- 1	Transient paresthesias	Center track 1	Center track (-)7-1	Transient paresthesias	Center track 1
5	VIM	Center track (-)6.5 – 1.5	Persistent paresthesias and mild speech slur @ 2.5mA	Center track 1.5	Center track (-)6.5- (-)1.5	Transient paresthesias and mild speech slur @ 2mA	Center track 1.5
6	VIM	Center track (-)9.5-(-)0.5	No side effects	Center track (-)0.5	Center track (-)9-(-)1	No side effects	Center track (-)1
7	VIM	Center track (-)5.5 – 1	No side effects	Center track 1.5	Center track (-)6.5 – 0	No side effects	Center track 0.5
8	VIM	Anterior track (-)6 – 1	No side effects	Anterior track 1	NA	NA	NA
9	VIM	Medial track(-)6 – 1	Transient paresthesias	Medial track 1	Center track (-)6.5- 1	Transient paresthesias	Center track 1
10	VIM	Center track (-)7-(-)1	No side effects	Center track (-)1	Center track (-)8-(-)0.5	Persistent paresthesia @3mA	Center track (-)0.5
11	VIM	Center track (-)3.5 – 1.5	No side effects	Center track 1.5	Center track (-)3.5 – 0	Transient paresthesias	Center track 0
12	VIM	Center track (-)5 – (-)0.5	Transient paresthesias	Center track (-)0.5	Center track (-)6-(-)1	Transient paresthesias	Center track (-)1
13	VIM	Center track (-)6.5 - (-)1	Persistent paresthesias @ 3mA	Center track (-)1	Center track (-)6.5 – 0	Persistent paresthesias @ 3mA	Center track 0

Statistical analysis

Descriptive statistics of the patient population, including age, gender, time from surgery to final programming, UPDRS Part III scores, LED scores, and FTM scores, were collected. The frequency of directional programming use in both the ET and PD populations was also recorded. Average current, pulse width, and stimulation frequencies of DBS programming parameters were noted for each group. A paired Student’s t-test was used to compare pre-surgical and post-surgical LED scores (alpha level set at 0.05). The Mann-Whitney U test was used to compare changes in pre-surgical and post-surgical UPDRS Part III and FTM scores (alpha level set at 0.05).

## Results

Parkinson's disease

Details of the patient population, including responses to stimulation based on UPDRS Part III scores and reductions in LED, are summarized in Table 2. All 12 patients received directional programming, with 3 patients (25%) receiving it unilaterally and 9 patients (75%) bilaterally. The most common reason for switching to directional programming was the presence of capsular side effects, reported in 7 patients (58% of directional programming events). One patient (8%) experienced autonomic symptoms described as facial flushing, while 3 patients (25%) had improved symptom control, including better management of toe curling, dyskinesia, and tremor. Average DBS programming parameters are presented in Table 3, and individual patient data are shown in Table 4. 

**Table 2:** Demographics for PD patients LED: levodopa equivalent dose, PD: Parkinson's disease.

	N	Average (standard deviation)	p (pre-surgical vs post-surgical)
Age	12	60.67 (9.59)	-
Gender			
Male	5		
Female	7		
Time from surgery to programming (months)	12	11.08 (6.84)	-
UPDRS part III motor score			0.004
Pre-surgical (on med)	12	27 (11.97)	
Post-surgical (on med/on stim)	12	15.25 (7.91)	
LED			0.003
Pre-surgical	12	1089.17 (388.36)	
Post-surgical	12	756.67 (343.56)	
Directional programming	12		
Bilateral	9		
Unilateral	3		

**Table 3:** DBS settings for Parkinson’s disease patients with bilateral STN implants *Four patients had interleaving (two different current settings) that were excluded from this calculation. **One patient had interleaving (two different current settings) that were excluded from this calculation. ***Two patients had interleaving with different pulse widths that were excluded from this calculation. DBS: deep brain stimulation, SD: standard deviation, STN: subthalamic nucleus.

DBS parameters	n	Average (SD) (right)	n	Average (SD) (left)
Current (milliamperes)	12	2.02 (0.61)**	11	1.91 (0.60)*
Frequency (hertz)	12	149.08 (16.35)**	13	153.61 (21.63)
Pulse width (microseconds)	12	71.67 (10.30)**	11	80 (15.49)*

**Table 4:** Individual data for PD patients DBS: deep brain stimulation, UPDRS: Unified Parkinson's Disease Rating Scale, LED: levodopa equivalent dose, STN: subthalamic nucleus.

Patient	Age	Gender	Pre-DBS UPDRS	Post-DBS UPDRS	LED pre-DBS	LED post-DBS	Right STN	Left STN	Reason for directional
Patient 1	66	M	41	33	800 mg	500 mg	C+11-, 1.95 mA, 176 Hz, 80 us	C+3-2A-, 2.1 mA, 164 Hz, 80 us	R: Better tremor control. L: Better tremor control
Patient 2	60	M	20	10	1800 mg	1200 mg	C+12-, 1.6 mA, 172 Hz, 60 us	C+2A-, 1.4 mA, 144 Hz, 60 us	R: N/A. L: Better symptom control
Patient 3	57	M	22	10	800 mg	500 mg	Interleaved: p1: C+12-, 3.15 mA, 182 Hz, 90 us. p2: C+12-10B-, 2.70 mA, 182 Hz, 90 us	C+3B-2C-, 2.8 mA, 166 Hz, 90 us	R: Avoidance of muscle contractions. L: Avoidance of muscle contractions
Patient 4	77	F	50	13	1075 mg	875 mg	C+11B-, 1.95 mA, 184 Hz, 60 us	C+3A-, 1.35 mA, 160 Hz, 90 us	R: Avoidance of capsular effects. L: N/A
Patient 5	63	M	18	7	875 mg	1275 mg	Interleaved: p1: C+11A-9-, 1.80 mA, 125 Hz, 90 us. p2: C+12-10A-, 1.50 mA, 125 Hz, 90 us	1+2A-2B-, 2 mA, 125 Hz, 90 us	R: N/A. L: Avoidance of stiffness (capsular effect)
Patient 6	50	F	18	15	1250 mg	1075 mg	C+11A-, 1.85 mA, 160 Hz, 70 us	C+2A-, 1.80 mA, 150 Hz, 60 us	R: Better symptom control. L: Better symptom control
Patient 7	73	F	21	15	600 mg	450 mg	C+10C-, 1.40 mA, 130 Hz, 60 us	3A+3B-3C-2A+2B-2C-1-, 2.00 mA, 188 Hz, 70 us	R: Avoidance of feeling flushed and hot. L: Avoidance of feeling flushed and hot
Patient 8	57	F	19	9	600 mg	200 mg	Interleaved p1: 11+10A-10B+10C-, 1.90 mA, 125 Hz, 100 us. p2: 11A-11B-11C-10A+, 2.10 mA, 125 Hz, 80 us	3C-2B-1+, 2.00 mA, 125 Hz, 80 us	R: Avoidance of pulling of eyes and mouth. L: Avoidance of pulling of eyes and mouth
Patient 9	57	M	42	18	1580 mg	1080 mg	12+11-10B-9+, 2.10 mA, 170 Hz, 60 us	C+2A-1-, 1.65 mA, 182 Hz, 70 us	R: Avoidance of capsular side effects. L: Avoidance of capsular side effects
Patient 10	64	F	30	8	1465 mg	650 mg	Interleaved: p1: 11A+11B+11C-10B-10C+9-, 2.05 mA, 125 Hz, 110 us. p2: 12+11B-11C+10A+, 2.15 mA, 125 Hz, 120 us	Interleaved: p1: 4-3A+3B-3C-2A+2B-2C-1+, 1.95 mA, 125 Hz, 90 us. p2: 4+3B-3C-2B-2C+1+, 2.10 mA, 125 Hz, 90 us	R: Avoidance of pulling of face and dysarthria. L: Avoidance of pulling of face and dysarthria
Patient 11	63	F	32	27	1275 mg	775 mg	12+11A-10B-, 2.15 mA, 188 Hz, 60 us	4+3A-3C-2B-, 2.30 mA, 188 Hz, 60 us	R: Avoidance of dexterity issues. L: Avoidance of dexterity issues
Patient 12	41	F	11	18	950 mg	500 mg	C+11A-, 2.0 mA, 156 Hz, 60 us	C+2-, 2.0 mA, 138 Hz, 60 us	R: Improvement of dyskinesia, hand dexterity, and toe curling. L: N/A

Essential tremor

Of the 13 ET patients included, 12 (92%) had bilateral VIM implants and 1 had a unilateral implant. Patient demographics and response to stimulation are detailed in Table 5, and average DBS settings are shown in Table 6. Directional stimulation was used bilaterally in five patients (39%) and unilaterally in six (46%), including the patient with a unilateral implant. Seven patients (64% of all directional programming events) were programmed with directional stimulation to avoid side effects such as capsular and sensory symptoms. Four patients (31%) received directional stimulation for improved tremor control, while one patient (8%) had directional programming to reduce side effects on one side and improve control on the other. Overall, 45% (5 of 11) of all directional programming decisions were made for improved therapeutic effect. Reported side effects included dysarthria (n = 4; 31%), sensory disturbances (n = 2; 16%), pulling of the mouth (n = 2; 16%), and eye deviation (n = 1; 8%), with individual data presented in Table 7.

**Table 5:** Demographics for ET patients FTM: Fahn-Tolossa-Marin, ET: essential tremor.

Demographics	N	Average (standard deviation)	p (pre-surgical vs post-surgical)
Age (years)	13	63.54 (8.27)	-
Gender			-
Male	9		
Female	4		
Time from surgery to programming (months)	13	11.08 (7.48)	-
FTM			0.008
Pre-surgical	12	62.25 (23.99)	
Post-surgical	9	13.56 (7.11)	
Directional programming	11		-
Bilateral	6		
Unilateral	5		

**Table 6:** DBS settings for essential tremor patients with bilateral VIM implants *Two patients had interleaved settings which were excluded from this analysis. **One patient had interleaved settings (two different parameters) which were excluded from this analysis. DBS: deep brain stimulation, SD: standard deviation, VIM: ventral intermediate nucleus.

DBS parameters	n	Average (SD) (right)	n	Average (SD) (left)
Current (milliamperes)	12	2.02 (0.61)**	11	1.91 (0.60)*
Frequency (hertz)	12	149.08 (16.35)**	13	153.61 (21.63)
Pulse width (microseconds)	12	71.67 (10.30)**	11	80 (15.49)*

**Table 7:** Individual data for ET patients VIM: ventral intermediate nucleus, ET: essential tremor.

Patient	Age	Gender	Right VIM	Left VIM	Reason for directional
Patient 1	73	M	C+10A-, 2.3 mA, 146 Hz, 70 us	C+1-, 2.6 mA, 190 Hz, 110 us	R: Better tremor control. L: N/A
Patient 2	71	M	C+11A-, 0.95 mA, 125 Hz, 70 us	Interleaved: p1: C+3A-2A-, 2.5 mA, 125Hz, 90 us. p2: 3A-2A-2B-2C-1+, 2.30 mA, 125 Hz, 90 us	R: N/A. L: Avoidance of dysarthria
Patient 3	71	F	C+2C-, 2.7 mA, 146 Hz, 80 us	Interleaved: p1: 4-3+, 3.50 mA, 124 Hz, 90 us. p2: 3+2B-2C-, 2.45 mA, 124 Hz, 80 us	R: Avoidance of pulling of mouth. L: Avoidance of pulling of mouth
Patient 4	60	M	11B-11C-9+, 1.6 mA, 142 Hz, 60 us	C+2A-, 1.5 mA, 138 Hz, 60 us	R: Avoidance of left hand heaviness, tingling lips/tongue, eyes pulling. L: N/A
Patient 5	50	F	C+11-, 1.6 mA, 166 Hz, 90 us	C+4-, 1.55 mA, 162 Hz, 90 us	R: N/A. L: N/A
Patient 6	74	M	C+11B-10B-, 2.60 mA, 180 Hz, 70 us	C+1-, 2.85 mA, 180 Hz, 80 us	R: Better tremor control. L: N/A
Patent 7	53	M	C+11B-10A-, 1.55 mA, 154 Hz, 70 us	C+2-, 1.45 mA, 146 Hz, 80 us	R: Better tremor control. L: N/A
Patient 8	55	M	N/A	3+2+1-, 0.95 mA, 158 Hz, 60 us	L: Avoidance of dysphagia and dysarthria
Patient 9	60	F	C+10-, 1.90 mA, 144 Hz, 60 us	C+2A-2B-, 1.75 mA, 156 Hz, 70 us	R: Better tremor control. L: Better tremor control
Patient 10	69	M	12+10B-, 1.45 mA, 164 Hz, 60 us	4+2C-, 1.45 mA, 158 Hz, 70 us	R: Avoidance of dysarthria. L: Avoidance of dysarthria
Patient 11	65	F	11A+10A-, 2.00 mA, 130 Hz, 70 us	4-3+2A-2B+2C-1-, 2.20 mA, 144 Hz, 80 us	R: Avoidance of dysarthria. L: Avoidance of dysarthria
Patient 12	69	M	11A-11B-11C-10A+, 2.80 mA, 160 Hz, 90 us	C+1-, 2.4 mA, 184 Hz, 80 us	R: Avoidance of tongue numbness. L: N/A
Patient 13	56	M	C+11A-10A-9-, 2.80 mA, 132 Hz, 70 us	C+3B-3C-, 2.35 mA, 132 Hz, 100 us	R: Better tremor control. L: Avoidance of facial pulling and better tremor control

## Discussion

After its introduction, directional DBS programming was rapidly incorporated into clinical practice at our institution. This aligns with findings from previous studies evaluating the use of directional stimulation. For example, Maciel et al. compared early versus delayed initiation of directional programming in a small cohort of PD patients (n = 13; 23 directional electrodes) and found increased use of directional settings over time, with a marked reduction in omnidirectional stimulation once directional electrodes were employed [11].

At our institute, DBS programming typically begins with a monopolar review of each contact, though subsegmental testing is not performed during the initial session. Directional programming was more commonly applied to reduce side effects, particularly internal capsule-related symptoms such as dysarthria, dysphagia, or involuntary contraction of contralateral muscles. In some cases, directional settings were used primarily to enhance symptom control. Previous studies have demonstrated improved therapeutic outcomes with directional versus omnidirectional stimulation in PD patients [12-14]. In one trial involving 10 patients with STN stimulation, directional programming led to better hand rotation compared to omnidirectional settings [14]. One proposed explanation is that the smaller surface area of directional contacts results in higher charge density at the same current amplitude, thereby improving clinical efficacy [6].

Other studies have demonstrated a wider therapeutic window and reduced current requirements with directional programming in ET patients [15]. Similar benefits have been reported in PD patients, as seen in the PROGRESS trial, where 91% of participants experienced an improved therapeutic window with directional stimulation [16].

One challenge associated with directional leads is managing the rotational degree of freedom during implantation. Twisting of the flexible leads can result in deviations of up to 30 degrees from the intended orientation. To minimize the impact of such rotation, meticulous surgical technique is essential. Postoperative delayed rotational fluoroscopy may help assess deviations from the expected lead orientation [6,17]. Additionally, advanced targeting methods, such as anatomy-based planning and non-contrast imaging protocols, may further enhance accuracy and optimize outcomes [18,19].

Our study has several limitations, primarily stemming from its retrospective design and single-center setting. The absence of a comparison cohort within our own practice, along with the relatively small sample size, limits the ability to draw definitive conclusions about the comparative value of directional versus non-directional stimulation. However, our findings suggest that directional stimulation may be superior, as patients in our cohort, acting as their own controls, achieved improved therapeutic outcomes with directional settings. This hypothesis warrants evaluation in a prospective trial. That said, conducting such a trial may have limited utility, as non-directional leads are no longer offered by any manufacturer. Additionally, our cohort may not be fully representative, as the selection of device type was not blinded. Nonetheless, we believe the risk of selection bias is low, given that device choice was based on surgeon preference, while programming was performed independently by the treating neurologist. The programming was conducted by a single provider, which enhanced consistency in technique and clinical assessment. However, standardized scoring was not always feasible, resulting in some missing exam data in both PD and ET groups. Follow-up times also varied among patients, though the average duration of follow-up was approximately 11 months in both groups, allowing sufficient time for programming optimization. It is worth noting that subsegmental monopolar review was not performed during the initial programming visit; instead, directional stimulation of subsegments was implemented later based on clinical need.

## Conclusions

This study highlights the practical use of directional programming in a standard clinical setting. Our findings emphasize the potential benefit of incorporating directional stimulation during the initial programming visit, which may help reach optimal settings more quickly. By expanding the therapeutic window, directional programming can reduce side effects and improve overall quality of life. We believe that current steering has the potential to shift traditional DBS programming practices toward more precise and individualized care.
